# Complete genome sequence of *Tsukamurella paurometabola* type strain (no. 33^T^)

**DOI:** 10.4056/sigs.1894556

**Published:** 2011-06-30

**Authors:** A. Christine Munk, Alla Lapidus, Susan Lucas, Matt Nolan, Hope Tice, Jan-Fang Cheng, Tijana Glavina Del Rio, Lynne Goodwin, Sam Pitluck, Konstantinos Liolios, Marcel Huntemann, Natalia Ivanova, Konstantinos Mavromatis, Natalia Mikhailova, Amrita Pati, Amy Chen, Krishna Palaniappan, Roxanne Tapia, Cliff Han, Miriam Land, Loren Hauser, Yun-Juan Chang, Cynthia D. Jeffries, Thomas Brettin, Montri Yasawong, Evelyne-Marie Brambilla, Manfred Rohde, Johannes Sikorski, Markus Göker, John C. Detter, Tanja Woyke, James Bristow, Jonathan A. Eisen, Victor Markowitz, Philip Hugenholtz, Nikos C. Kyrpides, Hans-Peter Klenk

**Affiliations:** 1 DOE Joint Genome Institute, Walnut Creek, California, USA; 2 Los Alamos National Laboratory, Bioscience Division, Los Alamos, New Mexico, USA; 3 Biological Data Management and Technology Center, Lawrence Berkeley National Laboratory, Berkeley, California, USA; 4 Oak Ridge National Laboratory, Oak Ridge, Tennessee, USA; 5 HZI – Helmholtz Centre for Infection Research, Braunschweig, Germany; 6 DSMZ - German Collection of Microorganisms and Cell Cultures GmbH, Braunschweig, Germany; 7 University of California Davis Genome Center, Davis, California, USA; 8 Australian Centre for Ecogenomics, School of Chemistry and Molecular Biosciences, The University of Queensland, Brisbane, Australia

**Keywords:** obligately aerobic, non-motile, mesophilic, chemoorganotrophic, Gram-positive, metachromatic granules, opportunistic pathogen, *Tsukamurellaceae*, GEBA

## Abstract

*Tsukamurella paurometabola* corrig. (Steinhaus 1941) Collins *et al.* 1988 is the type species of the genus *Tsukamurella*, which is the type genus to the family *Tsukamurellaceae*. The species is not only of interest because of its isolated phylogenetic location, but also because it is a human opportunistic pathogen with some strains of the species reported to cause lung infection, lethal meningitis, and necrotizing tenosynovitis. This is the first completed genome sequence of a member of the genus *Tsukamurella* and the first genome sequence of a member of the family *Tsukamurellaceae*. The 4,479,724 bp long genome contains a 99,806 bp long plasmid and a total of 4,335 protein-coding and 56 RNA genes, and is a part of the *** G****enomic* *** E****ncyclopedia of* *** B****acteria and* *** A****rchaea * project.

## Introduction

Strain no. 33^T^ (= DSM 20162 = ATCC 8368 = JCM 10117) is the type strain of the species *Tsukamurella paurometabola*, which in turn is the type species of the genus *Tsukamurella* [1,2]. Currently, there are eleven species within the genus *Tsukamurella* [1,3], which is named in honor of Michio Tsukamura, a Japanese microbiologist [1]. The species epithet derives from the Greek words *paurus* meaning *little* and *metabolus* meaning *changeable*, referring to a metabolism that is little changeable [1]. Strain no. 33^T^ was first isolated from the mycetome and ovaries of *Cimex lectularis* (bedbug) in a study on the bacterial flora of *Hexapoda* by Edward A. Steinhaus in 1941 [2]. *T. paurometabola* was formerly also known as *Corynebacterium paurometabolum* (basonym) [1,4] as well as under its heterotypic synonym *Rhodococcus aurantiacus* [5,6], until Collins *et al.* revised the controversial taxonomic position of the species in 1988 [1] and J. P. Euzéby corrected the species epithet according to the rules of to the International Code of Nomenclature of Bacteria (1990 Revision) [7]. *T. paurometabola* is known, albeit rarely, to be an opportunistic pathogen for humans, especially in patients with predisposing conditions, such as immunosuppression (leukemia, solid tumors, and HIV infection) [8,9], chronic lung disease (tuberculosis) [9], and most often indwelling foreign bodies (long-term use of indwelling catheters) [10-13]. Here we present a summary classification and a set of features for *T. paurometabola* no. 33^T^, together with the description of the complete genomic sequencing and annotation.

## Classification and features

**Figure 1:**
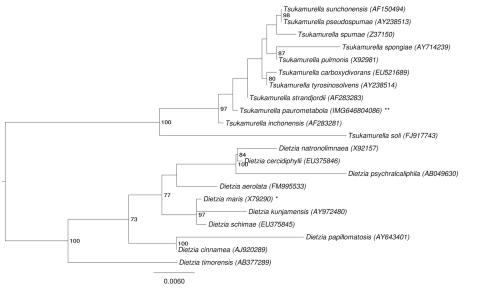
Phylogenetic tree highlighting the position of *T. paurometabola* relative to the other type strains within the genus *Tsukamurella*. The tree was inferred from 1,447 aligned characters [14,15] of the 16S rRNA gene sequence under the maximum likelihood criterion [16] and rooted with the members of the closely related genus *Dietzia*. The branches are scaled in terms of the expected number of substitutions per site. Numbers above branches are support values from 1,000 bootstrap replicates [17] if larger than 60%. Lineages with type strain genome sequencing projects registered in GOLD [18] are labeled with one asterisk, those registered as 'Complete and Published' with two asterisks.

The phylogenetic neighborhood of *T. paurometabola* no. 33^T^ in a 16S rRNA based tree is shown in Figure 1. The sequences of the two identical 16S rRNA gene copies in the genome differ by one nucleotide from the previously published 16S rRNA sequence (AF283280).

A representative genomic 16S rRNA sequence of strain no. 33^T^ was compared using NCBI BLAST under default settings (e.g., considering only the high-scoring segment pairs (HSPs) from the best 250 hits) with the most recent release of the Greengenes database [19] and the relative frequencies, of taxa and keywords (reduced to their stem [20]) were determined, weighted by BLAST scores. The most frequently occurring genera were *Tsukamurella* (34.7%), *Mycobacterium* (32.5%), *Dietzia* (20.6%) and *Rhodococcus* (12.1%) (220 hits in total). Regarding the seven hits to sequences from members of the species, the average identity within HSPs was 99.3%, whereas the average coverage by HSPs was 96.7%. Regarding the 45 hits to sequences from other members of the genus, the average identity within HSPs was 99.2%, whereas the average coverage by HSPs was 96.2%. Among all other species, the one yielding the highest score was *Tsukamurella strandjordii*, (NR_025113), which corresponded to an identity of 99.5% and a HSP coverage of 100.0%. (Note that the Greengenes database uses the INSDC (= EMBL/NCBI/DDBJ) annotation, which is not an authoritative source for nomenclature or classification.) The highest-scoring environmental sequence was DQ366095 ('on Oil Degrading Consortium oil polluted soil clone MH1 Pitesti'), which showed an identity of 99.2% and an HSP coverage of 99.0%. The most frequently occurring keywords within the labels of environmental samples which yielded hits were 'skin' (9.6%), 'human' (4.8%), 'microbiom, tempor, topograph' (4.2%), 'sea' (3.8%) and 'sediment' (1.8%) (30 hits in total). Environmental samples which yielded hits of a higher score than the highest scoring species were not found. These environmental labels are in line with the locations reported for the isolation of *Tsukamurella* strains, such as soil, human sputum, and bed bug [2,21].

**Figure 2:**
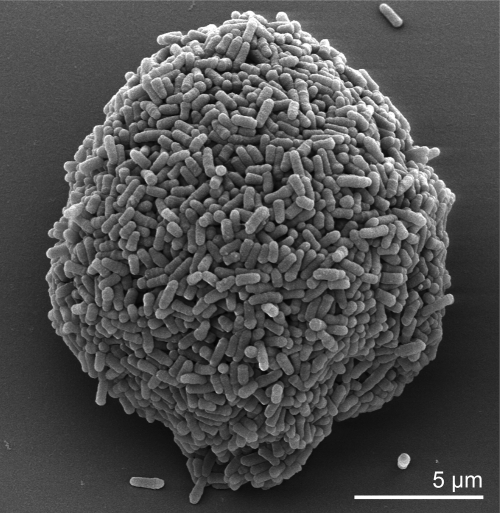
Scanning electron micrograph of *T. paurometabola* no. 33^T^

The cells of *T. paurometabola* are straight to slightly curved rods with a size of 0.5-0.8 × 1.0-5 µm and occur singly, in pairs, or in masses [2,21] (Figure 2). The organism is Gram-positive, weakly acid-fast (some strains are strongly acid-fast), non-sporeforming and non-motile [2,21] (Table 1). The organism contains metachromatic granules [2]. Colonies of *T. paurometabola* are small (diameter, 0.5-2.0 mm) with convex elevation, have entire edges (sometimes rhizoidal), are dryish but easily emulsified and are white to creamy to orange in color [3.15]. *T. paurometabola* is strictly aerobic and chemoorganotrophic bacterium [1]. Reaction is positive for catalase and pyrazinamidase [1]. Acid is produced from some sugars [1]. The organism does not produce nitriles from nitrates [2]. Indole is not produced by *T. paurometabola* [2]. The organism is non-pathogenic for guinea pigs [2]. In general *T. paurometabola* strains grow in the range 10°C to 35°C. Strain no. 33^T^ does not grow at 45°C [1]. The strain did not survive heating at 60°C for 15 minutes [1]. Some strains of *T. paurometabola* produce acid from fructose, galactose, glucose, glycerol, inositol, manitol, mannose, sorbitol, sucrose, and trehalose [1]. Acid is not produced from L-arabinose, L-rhamnose, or D-xylose [1]. Some strains of *T. paurometabola* grow on ethanol, fructose, galactose, glucose, inositol, mannitol, mannose, melizitose, sorbitol, sucrose, trehalose, xylose, *n*-butanol, isobutanol, 2,3-butylene glycol, propanol, propylene glycol, citrate, fumarate, malate, pyruvate, and succinate [1]. The organism does not grow on adonitol, arabinose, inulin, lactose, raffinose, or rhamnose [1]. Acetamide and nicotinamide are used as sole nitrogen sources but not benzamide [1]. Acetamide, glutamate, glucosamine, monoethanolamine, and serine are used as sole sources of carbon and nitrogen [1]. *T. paurometabola* is able to degrade Tween 20, Tween 40, Tween 60, and Tween 80, but not adenine, casein, or elastin [1]. Some strains of *T. paurometabolum* degrade xanthine and tyrosine [1]. The organism produces *β*-galactosidase and urease, but not arylsulfatase or *α*-esterase [1]. *T. paurometabolum* is resistant to ethambutol (5 µg/ml), 5-fluorouracil (20 µg/ml), mitomycin C (10 µg/ml), and picric acid (0.2% w/v) [1]. The organism is susceptible to bleomycin (5 µg/ml) [1].

**Table 1:** Classification and general features of *T. paurometabola* no. 33^T^ according to the MIGS recommendations [22] and the NamesforLife database [23]

MIGS ID	Property	Term	Evidence code
	Current classification	Domain *Bacteria*	TAS [24]
		Phylum “*Actinobacteria*”	TAS [25]
		Class *Actinobacteria*	TAS [26]
		Subclass *Actinobacteridae*	TAS [26,27]
		Order *Actinomycetales*	TAS [26-29]
		Suborder *Corynebacterineae*	TAS [26,27]
		Family *Tsukamurellaceae*	TAS [26,27]
		Genus *Tsukamurella*	TAS [1]
		Species *Tsukamurella paurometabola*	TAS [1]
		Type strain no. 33	TAS [2]
	Gram stain	positive	TAS [2]
	Cell shape	short rods occurring singly, in pairs or in masses	TAS [2]
	Motility	none	TAS [2]
	Sporulation	none	TAS [2]
	Temperature range	10°C–35°C, not at 45°C	NAS [1]
	Optimum temperature	not reported	
	Salinity	not reported	
MIGS-22	Oxygen requirement	obligately aerobic	TAS [1]
	Carbon source	carbohydrates	TAS [1]
	Energy metabolism	chemoorganotroph	TAS [1]
MIGS-6	Habitat	soil, human sputum, insect microbiome	TAS [2,4]
MIGS-15	Biotic relationship	free-living	NAS
MIGS-14	Pathogenicity	infection of the lung, lethal meningitis, and necrotizing tenosynovitis	TAS [4]
	Biosafety level	1+	TAS [30]
	Isolation	ovaries of *Cimex lectularius* (bedbug)	TAS [2,4]
MIGS-4	Geographic location	most probably close to Columbus, Ohio	NAS
MIGS-5	Sample collection time	1941 or before	TAS [2]
MIGS-4.1	Latitude	not reported	
MIGS-4.2	Longitude	not reported	
MIGS-4.3	Depth	not reported	
MIGS-4.4	Altitude	not reported	

Evidence codes - IDA: Inferred from Direct Assay (first time in publication); TAS: Traceable Author Statement (i.e., a direct report exists in the literature); NAS: Non-traceable Author Statement (i.e., not directly observed for the living, isolated sample, but based on a generally accepted property for the species, or anecdotal evidence). These evidence codes are from of the Gene Ontology project [31]. If the evidence code is IDA, the property was directly observed by one of the authors or an expert mentioned in the acknowledgements.

### Chemotaxonomy

The major cell wall sugars of *T. paurometabola* are arabinose and galactose [1], but ribose and traces of glucose have also been observed (unpublished data, DSMZ). The diagnostic amino acid of peptidoglycan is *meso*-diaminopimelic acid (variation A1_γ_); the glycan moiety of cell walls contains *N*-glycolyl residues [1]. Arabinogalactan is covalently attached to the peptidoglycan [32]. Long-chain highly unsaturated mycolic acids (62 to 78 carbon atoms) are present and contain one to six double bonds [1]. Fatty acid esters released on pyrolysis of mycolic acids have 20 to 22 carbon atoms [1,21]. The major polar lipids of *T. paurometabola* are diphosphatidylglycerol, phosphatidylethanolamine, phosphatidylinositol, and mono- and diacylated phosphatidylinositol dimannosides [1,21]. Some strains of *T. paurometabola* produce glycolipids [1]. The long-chain cellular fatty acids are predominantly straight-chain saturated, mono-unsaturated, and 10-methyl branched acids [1]. Menaquinones are the sole respiratory quinones, with MK-9 predominating [1]: 80% MK-9 (H_0_), 6.8% MK-8 (H_0_), 3.5% MK-7 (H_0_), 2.3%.MK-10 (H_0_) and 6.7%.MK-8 (H_2_) (unpublished data, DSMZ).

## Genome sequencing and annotation

### Genome project history

This organism was selected for sequencing on the basis of its phylogenetic position [33], and is part of the *** G****enomic* *** E****ncyclopedia of* *** B****acteria and* *** A****rchaea * project [34]. The genome project is deposited in the Genome On Line Database [18] and the complete genome sequence is deposited in GenBank. Sequencing, finishing and annotation were performed by the DOE Joint Genome Institute (JGI). A summary of the project information is shown in Table 2.

**Table 2:** Genome sequencing project information

**MIGS ID**	**Property**	**Term**
MIGS-31	Finishing quality	Finished
MIGS-28	Libraries used	Three genomic libraries: Sanger 8 kb pMCL200 library, 40 kb (fosmid, pcc1Fos) library, 454 pyrosequence standard library
MIGS-29	Sequencing platforms	ABI3730, 454 GS FLX Titanium
MIGS-31.2	Sequencing coverage	8.25 × Sanger; 37.9 × pyrosequence
MIGS-30	Assemblers	Newbler version 1.1.02.15, phrap
MIGS-32	Gene calling method	Prodigal 1.4, GenePRIMP
	INSDC ID	CP001966 (chromosome) CP001967 (plasmid Tpau01)
	Genbank Date of Release	May 17, 2010
	GOLD ID	Gc01341
	NCBI project ID	29399
	Database: IMG-GEBA	646564587
MIGS-13	Source material identifier	DSM 20162
	Project relevance	Tree of Life, GEBA

### Growth conditions and DNA isolation

*T. paurometabola* no. 33^T^, DSM 2016, was grown in medium 535 (Trypticase soy broth medium) [35] at 28°C. DNA was isolated from 0.5-1 g of cell paste using MasterPure Gram Positive DNA Purification Kit (Epicentre MGP04100) following the standard protocol as recommended by the manufacturer, with modification st/LALMice for cell lysis as described in [24]. DNA is available through the DNA Bank Network [36].

### Genome sequencing and assembly

The genome was sequenced using a combination of Sanger and 454 sequencing platforms. All general aspects of library construction and sequencing can be found at the JGI website [37]. Pyrosequencing reads were assembled using the Newbler assembler (Roche). Large Newbler contigs were broken into 4,920 overlapping fragments of 1,000 bp and entered into assembly as pseudo-reads. The sequences were assigned quality scores based on Newbler consensus q-scores with modifications to account for overlap redundancy and adjust inflated q-scores. A hybrid 454/Sanger assembly was made using the parallel phrap assembler [38]. Possible mis-assemblies were corrected with Dupfinisher or transposon bombing of bridging clones [39]. Gaps between contigs were closed by editing in Consed, custom primer walk or PCR amplification. A total of 516 Sanger finishing reads were produced to close gaps, to resolve repetitive regions, and to raise the quality of the finished sequence. The error rate of the completed genome sequence is less than 1 in 100,000. Together, the combination of the Sanger and 454 sequencing platforms provided 46.15 × coverage of the genome. The final assembly contains 42,170 Sanger reads and 745,985 pyrosequencing reads.

### Genome annotation

Genes were identified using Prodigal [40] as part of the Oak Ridge National Laboratory genome annotation pipeline, followed by a round of manual curation using the JGI GenePRIMP pipeline [41]. The predicted CDSs were translated and used to search the National Center for Biotechnology Information (NCBI) non-redundant database, UniProt, TIGR-Fam, Pfam, PRIAM, KEGG, COG, and InterPro databases. Additional gene prediction analysis and functional annotation was performed within the Integrated Microbial Genomes - Expert Review (IMG-ER) platform [42].

## Genome properties

**Figure 3:**
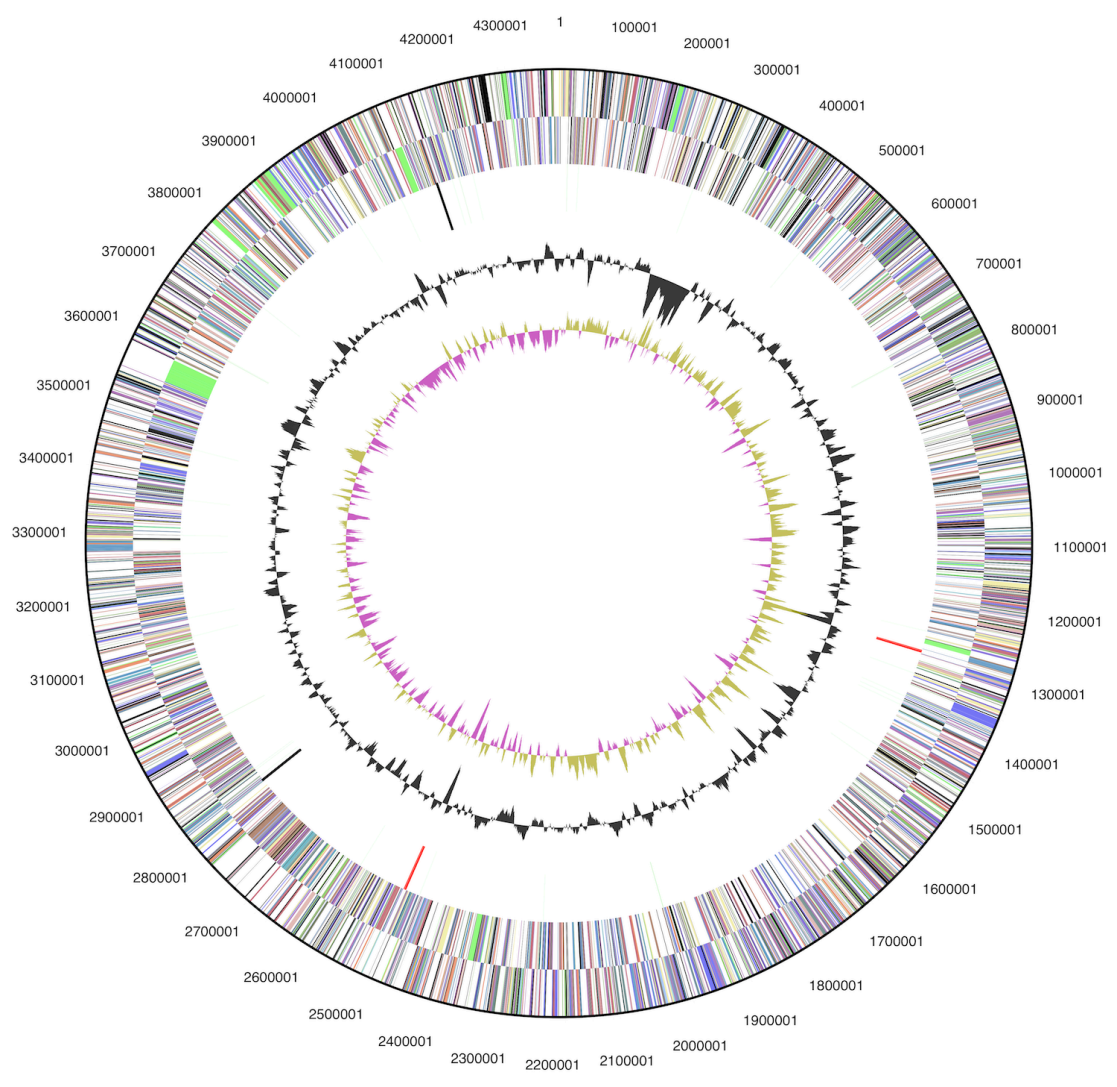
Graphical circular map of the chromosome. From outside to the center: Genes on forward strand (color by COG categories), Genes on reverse strand (color by COG categories), RNA genes (tRNAs green, rRNAs red, other RNAs black), GC content, GC skew.

The genome consists of a 4,379,918 bp long chromosome and a 99,806 bp long plasmid, both with a G+C content of 68.4% (Table 3 and Figure 3). Of the 4,391 genes predicted, 4,335 were protein-coding genes, and 56 RNAs; 93 pseudogenes were also identified. The majority of the protein-coding genes (68.7%) were assigned a putative function while the remaining ones were annotated as hypothetical proteins. The distribution of genes into COGs functional categories is presented in Table 4.

**Table 3:** Genome Statistics

**Attribute**	**Value**	**% of Total**
Genome size (bp)	4,479,724	100.00%
DNA coding region (bp)	4,108,044	91.70%
DNA G+C content (bp)	3,064,083	68.40%
Number of replicons	2	
Extrachromosomal elements	1	
Total genes	4,391	100.00%
RNA genes	56	1.28%
rRNA operons	2	
Protein-coding genes	4,335	98.72%
Pseudo genes	93	2.12%
Genes with function prediction	3,017	68.71%
Genes in paralog clusters	691	15.74%
Genes assigned to COGs	3,025	68.89%
Genes assigned Pfam domains	3,376	76.88%
Genes with signal peptides	1,031	23.48%
Genes with transmembrane helices	1,114	25.37%
CRISPR repeats	N.D.	

**Table 4:** Number of genes associated with the general COG functional categories

**Code**	**value**	**%age**	**Description**
J	169	5.0	Translation, ribosomal structure and biogenesis
A	1	0.0	RNA processing and modification
K	310	9.2	Transcription
L	198	5.9	Replication, recombination and repair
B	1	0.0	Chromatin structure and dynamics
D	31	0.9	Cell cycle control, cell division, chromosome partitioning
Y	0	0.0	Nuclear structure
V	39	1.2	Defense mechanisms
T	131	3.9	Signal transduction mechanisms
M	135	4.0	Cell wall/membrane/envelope biogenesis
N	3	0.1	Cell motility
Z	0	0.0	Cytoskeleton
W	0	0.0	Extracellular structures
U	29	0.9	Intracellular trafficking, secretion, and vesicular transport
O	102	3.0	Posttranslational modification, protein turnover, chaperones
C	217	6.4	Energy production and conversion
G	220	6.5	Carbohydrate transport and metabolism
E	274	8.1	Amino acid transport and metabolism
F	85	2.5	Nucleotide transport and metabolism
H	165	4.9	Coenzyme transport and metabolism
I	231	6.8	Lipid transport and metabolism
P	169	5.0	Inorganic ion transport and metabolism
Q	172	5.1	Secondary metabolites biosynthesis, transport and catabolism
R	430	12.7	General function prediction only
S	269	8.0	Function unknown
-	1,366	31.1	Not in COGs
